# Synaptic alterations are preceding the axonal loss in optic atrophy of Wolfram syndrome mouse model

**DOI:** 10.3389/fnins.2026.1838257

**Published:** 2026-05-22

**Authors:** Venu Gurram, William An, Shrinivas Bimal, Fumihiko Urano

**Affiliations:** 1 Department of Medicine, Division of Endocrinology, Metabolism, and Lipid Research, Washington University School of Medicine, St. Louis, MO, United States; 2 Department of Pathology and Immunology, Washington University School of Medicine, St. Louis, MO, United States

**Keywords:** axonal loss, inner plexiform layer, optic atrophy, retinal ganglion cells, synaptic alterations, WFS1, Wolfram syndrome

## Abstract

**Background:**

Wolfram syndrome is a rare autosomal recessive disorder characterized by antibody-negative early-onset diabetes mellitus, optic atrophy, sensorineural hearing loss, arginine-vasopressin deficiency, and progressive neurodegeneration of the brainstem and cerebellum. It is caused primarily by pathogenic variants in the *WFS1* gene, which encodes a transmembrane endoplasmic reticulum–resident protein involved in the unfolded protein response and cellular calcium homeostasis. Although multiple rodent models of Wolfram syndrome have been developed and shown to exhibit visual defects, some studies have reported significant vision loss prior to any detectable axonal degeneration or myelin abnormalities, and the mechanisms underlying these early visual deficits remain poorly understood.

**Objective:**

Recent *in vitro* studies have demonstrated altered synaptic contacts and aberrant neurite morphology in *WFS1*-deficient cerebral organoids and human iPSC-derived neurons, respectively. These findings prompted us to investigate, for the first time *in vivo*, whether synaptic and dendritic abnormalities occur in the retina of *Wfs1* knockout mice.

**Methods:**

Using confocal microscopy, we examined retinal and optic nerve histology in *Wfs1* knockout mice at 4 and 7 months of age.

**Results:**

Our analysis reveals progressive synaptic alterations in the inner plexiform layer, driven by early presynaptic compartment failure. These changes represent the earliest detectable phenotype associated with vision loss in this model and precede overt axonal degeneration.

**Conclusion:**

These findings identify early synaptic preservation as a promising therapeutic target for vision loss in Wolfram syndrome.

## 1 Introduction

Wolfram syndrome is a rare life-threatening neurodegenerative disorder, with no treatment, inherited in an autosomal recessive manner, affecting multiple organs, resulting in early-onset antibody-negative diabetes mellitus, optic atrophy, sensorineural hearing loss, arginine-vasopressin deficiency, and cerebellar and brainstem degeneration with a prevalence of 1 in 160,000–770,000 (Barrett et al., 1995; Caruso et al., 2024; Rando et al., 1992; Urano, 2016). The life expectancy of the majority of patients ranges from 30 to 40 years and respiratory failure or dysphagia are the common causes of death (Urano, 2016; Lee et al., 2023).

Wolfram syndrome is mainly caused by homozygous or compound heterozygous pathogenic variants in the WFS1 gene encoding a transmembrane protein localized to the endoplasmic reticulum (ER), wolframin (Inoue et al., 1998). Wolframin is a 890-amino acid protein with a molecular mass of 100 kDa, with its C-terminus oriented towards the ER lumen and its N-terminus towards the cytoplasm, and it can assemble and form tetramers (Inoue et al., 1998; Hofmann et al., 2003; Zatyka et al., 2008). WFS1 has been shown to play roles in the unfolded protein response, cellular calcium regulation, mitochondrial-associated membranes, and ER vesicular cargo export (Angebault et al., 2018; Osman et al., 2003; Takeda et al., 2001; Wang et al., 2021; Yamada et al., 2006; Fonseca et al., 2005; Fonseca et al., 2010; Lu et al., 2014).

Optic atrophy is of serious concern in patients with Wolfram syndrome due to its progressive nature. Case reports on patients with Wolfram syndrome have revealed progressive loss of visual acuity, color vision, and cecocentral scotomas starting in their early second decade of life, indicating the retinal ganglion cells (RGCs) as the primary pathological site (Seynaeve et al., 1994; Mtanda et al., 1986; Hoekel et al., 2014; Hoekel et al., 2018). Several research groups have studied optic atrophy in Wolfram syndrome using animal models. One study with the *Wfs1*-knockout model reported abnormal retinal function and slower conduction along the visual pathways with no RGC loss but with reduced axonal density compared to wildtype controls at 12 months of age (Bonnet Wersinger et al., 2014). Another study showed a significant reduction in the ratio of retinal thickness to longitudinal diameter and a decreased number of GFAP-positive cells in the inner nuclear layer (INL) as well as reduced GFAP intensity in the retina of *Wfs1* knockout mice compared to control mice at approximately 3.5 months of age (Waszczykowska et al., 2020). Recently, a study reported visual impairments, including reduced visual acuity, axonal loss, myelin degeneration, and increased retinal gliosis, but no RGC loss at 12 months of age in *Wfs1*-knockout mice (Rossi et al., 2023). In another study, progressive visual defects were reported starting as early as 3 months of age and myelin defects at 7.5 months in *Wfs1* knockout mice (Ahuja et al., 2024). More recently, two ex vivo magnetic resonance imaging (MRI) studies reported a significant reduction of optic nerve volume in *Wfs1*-deficient mice at 8 and 12 months of age compared with wild-type controls (Cagalinec et al., 2016; Tulva et al., 2025). A *Wfs1*-knockout rat model showed reduced visual acuity starting at 8.5 months, and axonal loss and increased retinal gliosis at 14–15 months, with significant RGC loss at 17–18 months (Seppa et al., 2019; Plaas et al., 2017; Jagomae et al., 2021). In a zebrafish *wfs1*-knockout model, significant RGC loss was observed at 4 months and a thinner GCL at 12 months, with significantly reduced visual acuity at both ages (Cairns et al., 2021).

A recent study showed disrupted synapses and altered neurite outgrowth in *Wfs1*-deficient cerebral organoids compared to control organoids (Yuan et al., 2023). Another study showed that primary cortical neurons transfected with *Wfs1*-shRNA exhibited a significant decrease in synaptic density at DIV19, but not at earlier stages (Cagalinec et al., 2016). Additionally, altered neurite outgrowth was also observed in human iPSC-derived neurons deficient for *Wfs1* (Pourtoy-Brasselet et al., 2021). It is therefore necessary to investigate synaptic and dendritic alterations *in vivo* in the Wolfram syndrome model. Although multiple previous studies have investigated optic atrophy in Wolfram syndrome, none have focused on synaptic and dendritic arborization in the retina of a Wolfram syndrome mouse model. Therefore, this study aims to provide comprehensive, age-dependent phenotyping of RGCs by examining their cell bodies in the retina and their axons in the optic nerve in a *Wfs1*-knockout mouse model of Wolfram syndrome.

## 2 Materials and methods

### 2.1 Animals

The 129S6 mice lacking *Wfs1* expression throughout the body were originally provided by Dr. Sulev Kõks at the University of Tartu (Koks et al., 2009; Luuk et al., 2009). In this strain, the segment of the WFS1 protein spanning amino acids 360 to 890 was substituted with an NLS-LacZ-Neo cassette inserted in frame. Genotyping was carried out using multiplex PCR performed by Transnetyx (Cordova, TN). All procedures involving animals followed the guidelines approved by the Washington University School of Medicine IACUC (Protocol #20-0334). Mice were housed in a specific pathogen-free facility in groups of 2–5 per cage under a 12 h light/12 h dark cycle with ad libitum access to food and water. Standard bedding and environmental enrichment with nesting material were provided.

This study used only male mice for all experiments and included in two age groups - 4 and 7 months. We selected 7-month-old mice as the upper age limit for this study because a significant number of *Wfs1* knockout mice begin to lose their eyes at around 8–9 months of age. For all histological experiments, we used a minimum of 5 animals per genotype. The sample sizes used for each analysis were as follows: Retinal ganglion cell quantification was performed using Brn3a- and RBPMS-labeled retinas (Wild-type (WT) = 7, *Wfs1* knockout = 6). Dendritic analysis was assessed at 4 months (WT = 7, *Wfs1* knockout = 6) and 7 months (WT = 6, *Wfs1* knockout = 5). Synaptic analysis included 4-month (WT = 6, *Wfs1* knockout = 7) and 7-month (WT = 7, *Wfs1* knockout = 6) cohorts. Retinal GFAP expression was evaluated at 4 months (WT = 7, *Wfs1* knockout = 6) and 7 months (WT = 6, *Wfs1* knockout = 5). Optic nerve analyses for GFAP, NF200, and MBP were conducted at 4 months (WT = 7, *Wfs1* knockout = 6) and 7 months (WT = 7, *Wfs1* knockout = 5).

### 2.2 Tissue collection and preparation

Mice were euthanized in a CO_2_ chamber for 7 min, and eyes and optic nerves were collected immediately. For RNA and protein analysis, tissues were flash-frozen and stored at −80 °C until use. For histology, optic nerves and eye cups (following removal of the cornea and lens) were fixed overnight at 4 °C in 4% paraformaldehyde. Tissues were then cryoprotected in 30% sucrose in PBS at 4 °C overnight. Eyes were subsequently embedded in OCT and stored at −20 °C. Optic nerves were embedded in gelatin as previously described (Cwerman-Thibault et al., 2017) and stored at −80 °C.

### 2.3 Immunohistochemistry and imaging

Retinas and optic nerves were sectioned at 10 μm using a cryostat (Leica CM1950) and mounted on SuperFrost Plus slides (12-550-15, Fisherbrand). Following PBS rinsing, sections were permeabilized with 0.25% Triton X-100 for 20 min at room temperature and blocked for 2 h in PBS containing 3% BSA, 5% goat serum, and 0.1% Triton X-100. Primary antibodies against Brn3a (MAB1585, Millipore, 1: 250, RRID: AB_94166), RBPMS (GTX118619, GeneTex, 1: 200, RRID: AB_10720427), NF200 (N4142, Sigma-Aldrich, 1: 2000, RRID: AB_477272), GFAP (PA1-10004, Invitrogen, 1: 2000, RRID: AB_1074620), MBP aa82–87 (MCA409S, Bio-Rad, 1: 50, RRID: AB_325004), *β*-III Tubulin (ab18207, Abcam, 1:750, RRID: AB_444319), PSD95 (51–6,900, Invitrogen, 1:150, RRID: AB_2533914), and Synaptophysin (101004, Synaptic Systems, 1:2000, RRID: AB_1210382) were applied overnight at 4 °C. Secondary antibodies (1:1000) and DAPI (0.5 μg/mL) were applied for 1 h at room temperature. For MBP staining, optic nerve sections were incubated with ice-cold methanol for 7 min at −20 °C prior to the permeabilization step.

For quantification of RGC densities, retinal sections labeled with anti-RBPMS and anti-Brn3a antibodies were imaged using tile scan on a Leica widefield microscope with 20 × objective to capture complete retinal sections at pixel resolution of 2.78 pixels/μm. Images were acquired from middle sections of the retina, avoiding peripheral sections. Cell quantification was performed using the Fiji cell counter plugin, a manual counting tool. Brightness and contrast settings were adjusted as needed for visual clarity during manual counting. The analyst was blinded to genotype during image acquisition and analysis. RGC number of each section is normalized to the length of corresponding Ganglion cell layer (GCL). Three to four retinal sections per mouse were evaluated for each genotype. Values corresponding to each retinal sections were averaged to yield a single data point per animal, which was used as the experimental unit in statistical analyses.

Imaging of retinal sections (for dendritic, synaptic, and GFAP analyses) was performed on a Nikon AXR confocal laser-scanning microscope using a PLAN APO λD 60x OIL OFN25 DIC N2 objective with a z-step size of 0.3 μm and a zoom factor of 2.0 at pixel resolution of 7.14 pixels/μm pixel size was 0.14 μm. Consistent imaging settings were maintained across all samples within each age-specific experiment. The Airy unit for the 488-nm (green) channel, used for PSD95 and *β*-III tubulin imaging, was 0.65 AU, while the Airy unit for the 594-nm (magenta) channel, used for SYP and GFAP imaging, was 0.56 AU. On average, 12 images were acquired per mouse, with four images collected from each of at least three retinal sections. Only middle retinal sections were imaged, focusing on the central retina and excluding peripheral regions and the optic nerve head.

Imaging of optic nerve sections (for axonal, GFAP and MBP analyses) was performed on a Zeiss LSM 880 confocal microscope equipped with Airyscan detector using tile scan (2 × 2) with a Plan-Apochromat 40×/1.3 Oil objective, in FastAiryScan mode, with z-step size of 0.22 μm and a zoom factor of 1.8, resulting in a pixel resolution of 11.1 pixels/μm pixel size was 0.09 μm. In FastAiryScan mode, these settings yielded an effective pinhole size of approximately 1.3 Airy Units. Identical imaging settings were maintained across all images within each age-specific experiment. We acquired one image per optic nerve section, with a minimum of 3 images per mouse. Each image captured almost the full extent of the optic nerve section.

### 2.4 Image analysis

Image analysis was performed using Imaris (version 10.2 or later) (Bitplane). Prior to quantification, all images underwent background subtraction followed by application of a consistent threshold cutoff. These preprocessing steps were applied uniformly across all images within each experiment to ensure comparability. Depending on the analysis type, either spots or surfaces were generated in Imaris using identical parameter settings for all samples.

For NF200-positive axonal counts, the Imaris Spots module was used to create and automatically quantify spot objects. For measurements of fluorescence intensity and volume, the Surface module was used to generate 3D surface reconstructions. Colocalization analyses were performed using the surface–surface colocalization function within Imaris.

For quantification of NF200 staining area, ImageJ was used. All z-stacked images were processed as maximum intensity projections, then converted to 8-bit images, and analyzed after applying a consistent threshold across all samples to calculate the fraction of NF200- positive staining area.

### 2.5 Immunoblot

Total protein lysates were isolated from retinal tissues using T-PER Tissue Protein Extraction Reagent (78510, ThermoFisher Scientific) supplemented with 1 × complete protease inhibitor cocktail (11873580001, MilliporeSigma). Protein lysates were mixed with 4 × Laemmli buffer (1610747, Bio-Rad) and heated at 45 °C for 25 min. Proteins were separated by SDS-PAGE and transferred to a Nitrocellulose Membrane (pore size 0.2 μm; 1620112, Bio-Rad). Primary antibodies used were anti-WFS1 (11558-1-AP, Proteintech, 1:1000, RRID: AB_2216046) and *β*-actin (4,967, Cell Signaling Technology, 1:3000, RRID: AB_330288). Secondary antibodies conjugated to horseradish peroxidase were used for detection. Protein bands were developed with ECL Select (RPN2235, MilliporeSigma) and imaged on a ChemiDoc MP Imaging System (Bio-Rad).

### 2.6 Quantitative real-time PCR

Total RNA was extracted from retinal tissue using the RNeasy Mini Kit (74106, Qiagen) according to the manufacturer’s instructions. RNA purity and concentration were assessed by spectrophotometry (A260/280 and A260/230 ratios) using NanoDrop 2000 (ND-2000, ThermoFisher). For each sample, 1 μg of RNA was reverse-transcribed using the SuperScript III First-Strand Synthesis System (18080051, Invitrogen) with oligo-dT primers in a total volume of 20 μL.

qPCR reactions were performed using PowerUp SYBR Green Master mix (A25742, Applied Biosystems) on a ViiA 7 Real-Time PCR system (Applied Biosystems, serial number: 278881187) with the following cycling conditions: denaturation at 95 °C for 15 s, annealing/extension at 60 °C for 60 s, and 40. Fluorescence thresholds were set automatically/manually and kept constant across plates. No-template controls were included in every run. Gene expression was normalized to the reference gene, *Gapdh*. Relative expression levels were calculated using the ΔΔCt method, and all samples were run in technical triplicate. Primer sequences used were: *Wfs1* (Forward: 5′-CCAGCTGAGGAACTTCAAGG-3′; Reverse: 5′-AGGATGACCACGGACAGTTC-3′) and *Gapdh* (Forward: 5′-TGTAGACCATGTAGTTGAGGTCA-3′; Reverse: 5′-AGGTCGGTGTGAACGGATTTG-3′).

### 2.7 Statistical analysis

All data were analyzed using GraphPad Prism. Data are presented as mean ± SEM relative to wild-type values unless otherwise noted. Comparisons between *Wfs1* knockout and WT mice were conducted using unpaired Student’s t-tests with Welch’s correction or Mann–Whitney tests, based on the Shapiro–Wilk test normality test results. The specific statistical test and *p* value for each individual result are detailed in the corresponding figure legend.

## 3 Results

### 3.1 No expression of Wfs1 protein and reduced body weight in Wfs1 knockout mice

**Figure 1:**
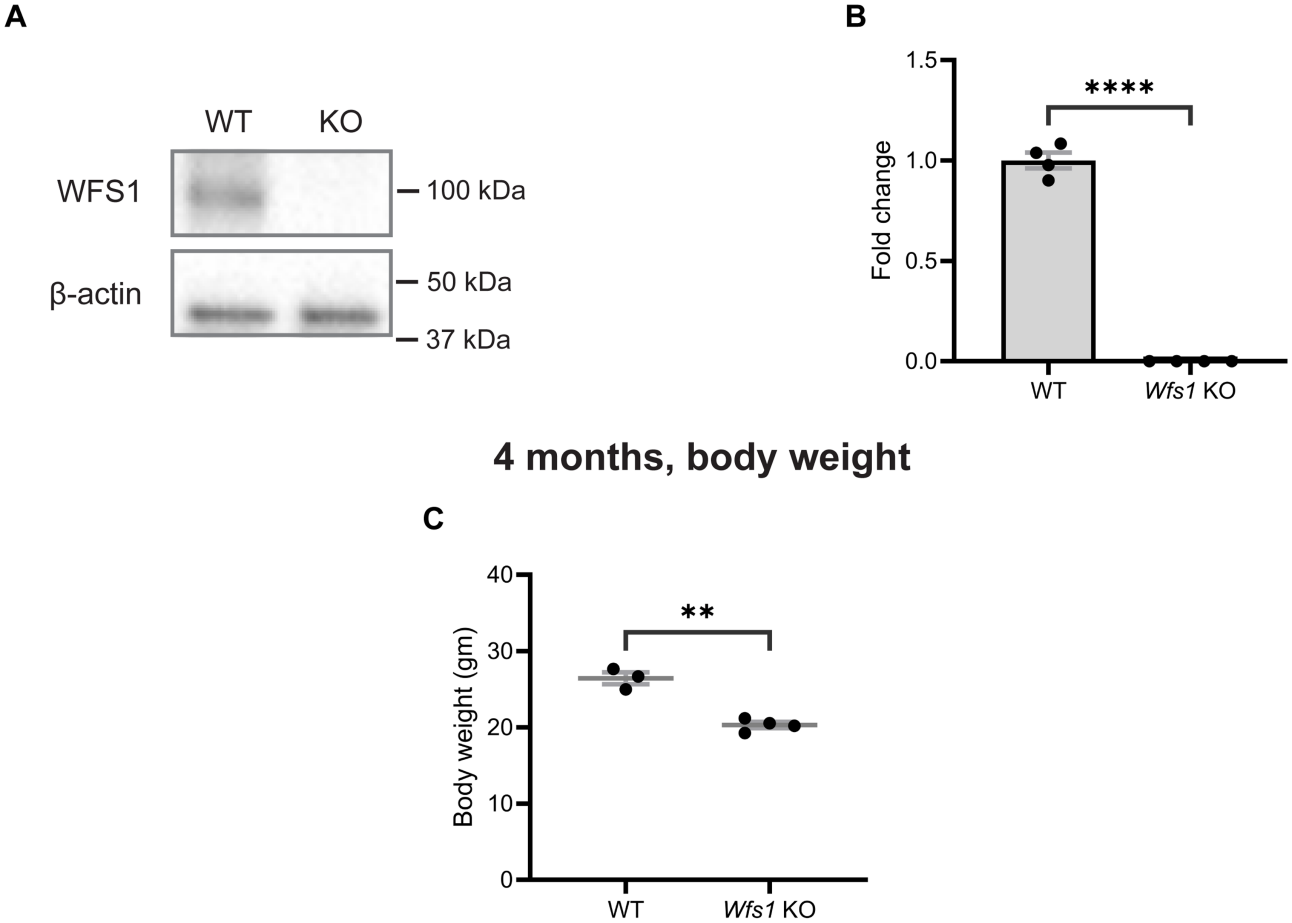
No wolframin expression and reduced body weight in *Wfs1* KO mice. **(A)** Representative immunoblot showing WFS1 expression in total protein lysate from retina of both wild type and *Wfs1* KO mice. B-actin was used as loading control. **(B)** Quantification of mRNA expression of *Wfs1* from total RNA from retina of wild type and *Wfs1* KO mice. Data are presented as normalized values ± SEM relative to WT mean. Statistical analysis was performed by two-tailed, unpaired Student’s *t*-test with Welch’s correction. *n* = 4. ****p < 0.0001. *Gapdh* as housekeeping gene. **(C)** Quantification of body weight of Wfs1 KO mice compared to the wild type control mice at the age of 4 months. Data are presented as mean ± SEM. Statistical analysis was performed by two-tailed, unpaired Student’s t-test with Welch’s correction. *n* ≥ 3 **p < 0.01.

*Wfs1* knockout mice were generated using a *Wfs1*-exon8 deletion, resulting in the complete knockout of WFS1 (Koks et al., 2009; Luuk et al., 2009). To confirm WFS1 deletion, western blot analysis was performed using whole retinal protein extract from 7-month-old animals. As expected, immunoblots showed no band in *Wfs1* knockout mice, whereas a clear band at 100 kDa was detected in WT controls (Figure 1A). Absence of expression was confirmed by qPCR (Figure 1B). As early as 4 months of age, male *Wfs1* knockout mice showed a statistically significant reduction in body weight compared to male wild-type littermates (Figure 1C). Body weight was not measured at earlier ages.

### 3.2 No RGC loss in Wfs1 knockout mice at 7 months of age

**Figure 2:**
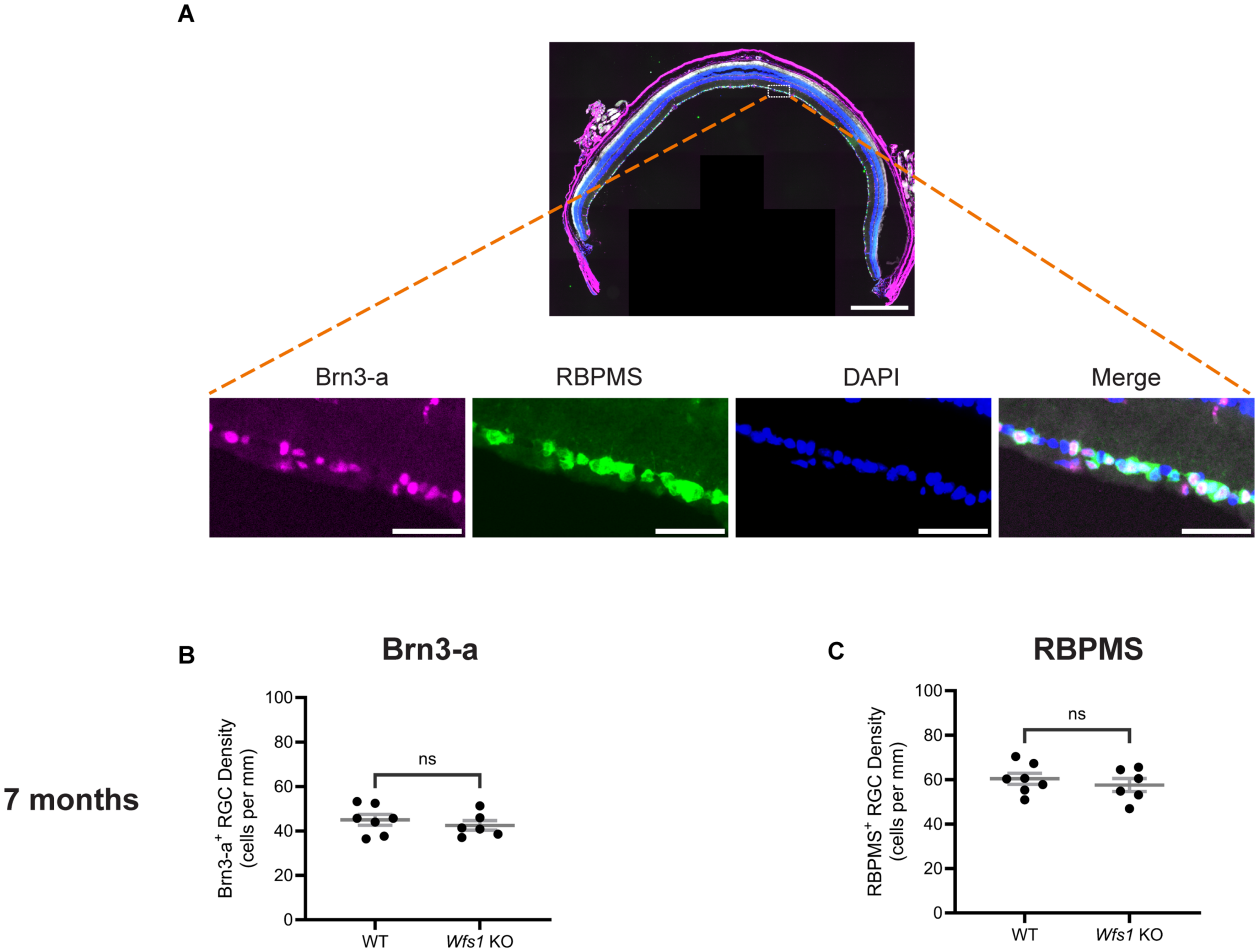
No RGC loss was evident in the Wfs1-KO model. **(A)** A representative mouse retinal section immunostained with both anti-Brn3-a (magenta) and anti-RBPMS (green) antibodies counterstained with DAPI (blue). Scale bar, 500 μm. High-magnification insets of the boxed area show the individual fluorescence channel and the merged overlay with a scale bar of 50 μm. Quantification of **(B)** Brn3a-positive RGC density **(C)** RBPMS-positive RGC density in both Wfs1 KO and control wild type mice of 7 month age. Quantification results were presented as mean ± SEM. Statistical analysis was performed by two-tailed, unpaired Student’s *t*-test with Welch’s correction. *n* ≥ 6. ns - not significant.

To evaluate RGC densities, mouse retinal cross-sections were stained with anti-Brn3a and anti-RBPMS, specific markers for RGC (Figure 2A). As previously reported, anti-RBPMS labels a greater number of RGCs than anti-Brn3a (Rodriguez et al., 2014). RGC density analysis showed no evidence of RGC loss in the GCL of *Wfs1* knockout mice compared with WT male littermates using either Brn3a^+^ (Figure 2B) or RBPMS^+^ (Figure 2C) analysis at 7 months of age. These results indicate that loss of *Wfs1* does not significantly reduce RGC density at 7 months of age, suggesting loss may occur at a more advanced stage in this knockout mouse model.

### 3.3 No sign of dendritic loss in Wfs1 knockout model

**Figure 3:**
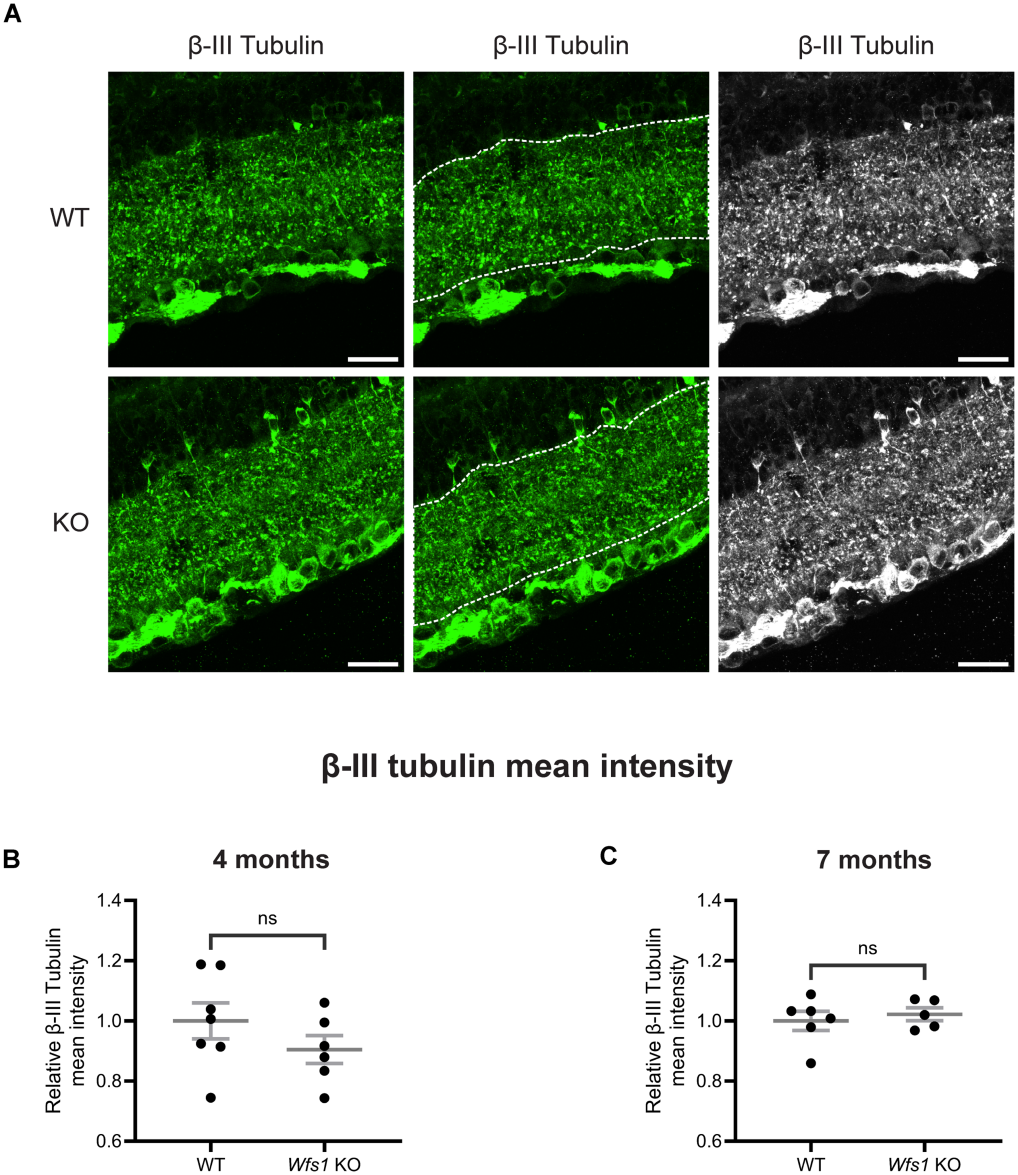
Dendritic architecture at gross level was preserved in Wfs1 KO mice at 4 and 7 months. **(A)** Retinal sections were immunolabeled with an anti-*β*-III tubulin antibody (green). Representative confocal images from 7-month-old WT (top) and Wfs1 KO (bottom) retinas are shown. Scale bar, 20 μm. Quantification of β-III tubulin fluorescence mean intensity in retinal sections from **(B)** 4-month and **(C)** 7-month old animals. Data are expressed as normalized values ± SEM relative to WT mean. Statistical comparisons were performed using a two-tailed unpaired Student’s *t*-test with Welch’s correction. *n* ≥ 4. ns - not significant.

To evaluate gross dendritic loss in the inner plexiform layer (IPL), retinal sections from WT and *Wfs1* knockout mice at 4 and 7 months of age were immunolabeled with anti-*β*-III tubulin, a marker of neuronal soma, dendrites, and axons (Cwerman-Thibault et al., 2017). *β*-III tubulin labeling in the IPL showed no difference between WT and *Wfs1* knockout mice at either age (Figure 3A). Quantification of β-III tubulin mean intensity showed no significant difference at 4 months (*Wfs1* knockout: 0.9048 ± 0.046 vs. WT: 1.000 ± 0.059; Figure 3B) or at 7 months (*Wfs1* knockout: 1.022 ± 0.021 vs. WT: 1.000 ± 0.031; Figure 3C). A similar pattern was observed with β-III tubulin staining volume, with no significant changes in *Wfs1* knockout mice compared to age- and sex-matched littermate controls, indicating no gross dendritic loss in the IPL.

### 3.4 Synaptic alterations are the earliest phenotype in Wfs1 knockout model

**Figure 4:**
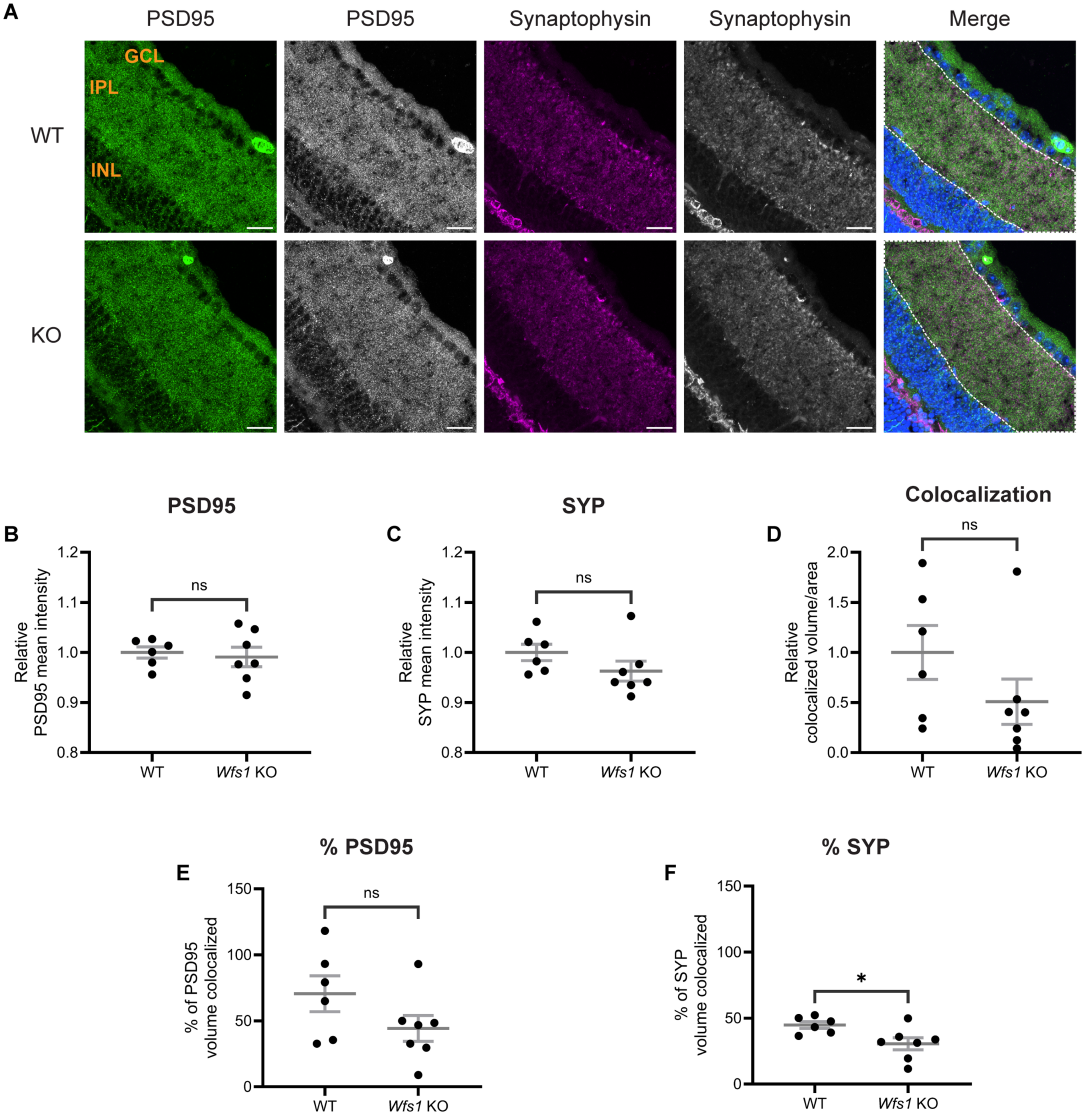
Altered synaptic connections were observed in Wfs1 KO retinas at 4 months. **(A)** Retinal sections were immunolabeled with an anti-PSD95 antibody (green) and anti-synaptophysin antibody (magenta). Representative confocal images from 4-month-old WT (top) and Wfs1 KO (bottom) mice are shown. Scale bar, 20 μm. Quantification of **(B)** PSD95 fluorescence mean intensity **(C)** synaptophysin fluorescence mean intensity in retinal sections from 4-month-old animals. **(D)** Quantification of relative colocalized volume of PSD95-synaptophysin per unit ROI area in 4-month-old mice. Data are presented as normalized values ± SEM relative to WT mean. Quantification of relative percentage of **(E)** PSD95 **(F)** synaptophysin colocalized volume in 4 month old mice. Data are presented as mean ± SEM. Statistical analysis was performed using a two-tailed unpaired Student’s *t*-test with Welch’s correction **(B,E,F)** Mann–Whitney test **(C,D)**. *n* ≥ 6. *p.

**Figure 5:**
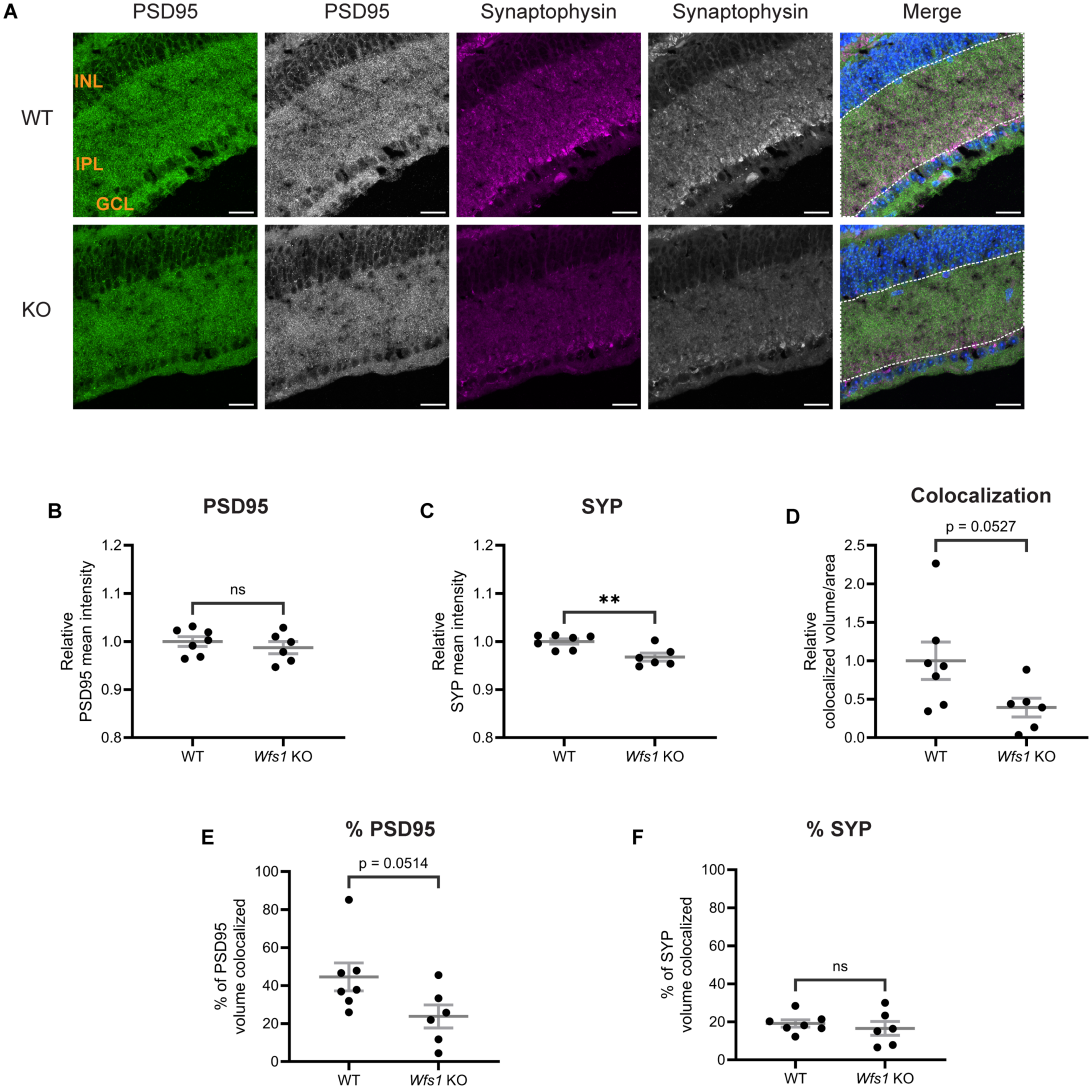
Synaptic loss was observed in Wfs1 KO retinas at 7 months. **(A)** Retinal sections were immunolabeled with an anti-PSD95 antibody (green) and anti-synaptophysin antibody (magenta). Representative confocal images from 7-month-old WT (top) and Wfs1 KO (bottom) mice are shown. Scale bar, 20 μm. Quantification of **(B)** PSD95 fluorescence mean intensity **(C)** synaptophysin fluorescence mean intensity in retinal sections from 7-month-old animals. **(D)** Quantification of relative colocalized volume of PSD95-synaptophysin per unit ROI area in 7-month-old mice. Data are presented as normalized values ± SEM relative to WT mean. Quantification of relative percentage of **(E)** PSD95 **(F)** synaptophysin colocalized volume in 7 month old mice. Data are presented as mean ± SEM. Statistical analysis was performed using a two-tailed unpaired Student’s *t*-test with Welch’s correction. *n* ≥ 6. **p.

To characterize the retinal phenotype further, we examined synaptic connections in the IPL, where RGCs form synapses with bipolar and amacrine cells via their dendrites (Yu et al., 2013). Retinal sections from WT and *Wfs1* knockout mice at 4 months (Figure 4A) and 7 months (Figure 5A) were immunostained with anti-PSD95 (postsynaptic density marker) and anti-synaptophysin (SYP; presynaptic marker).

At 4 months of age, quantification of PSD95 mean intensity showed no difference (WT: 1.000 ± 0.011; vs. *Wfs1* knockout: 0.9910 ± 0.019; Figure 4B). SYP mean intensity was also similar between genotypes (WT: 1.000 ± 0.016; *Wfs1* knockout: 0.9627 ± 0.019; Figure 4C). Quantification of staining volume of PSD95 and SYP showed similar results (Supplementary Figures S1A,B). Object-based colocalization of PSD95 and SYP showed no statistically significant difference in colocalized volume per unit area (WT: 1.000 ± 0.269 vs. *Wfs1* knockout: 0.5081 ± 0.226; Figure 4D). However, the percentage of SYP volume colocalized with PSD95 was significantly decreased in *Wfs1* knockout mice (WT: 44.80 ± 2.557 vs. *Wfs1* knockout 30.56 ± 4.612 vs.; *p* = 0.0239; Figure 4F), while no significant difference was observed in the percentage of PSD95 volume colocalized (*Wfs1* knockout: 44.23 ± 9.802 vs. WT: 70.60 ± 13.60; Figure 4E).

At 7 months of age, PSD95 mean intensity remained unchanged (WT: 1.000 ± 0.010 vs. *Wfs1* knockout: 0.9872 ± 0.012; Figure 5B). By contrast, SYP mean intensity was significantly decreased in *Wfs1* knockout mice (WT: 1.000 ± 0.005 vs. *Wfs1* knockout: 0.9677 ± 0.008; *p* = 0.009; Figure 5C). Similar results were observed with quantification analysis of staining volume of PSD95 and SYP (Supplementary Figures S1C,D). Colocalization analysis showed a 60% reduction in colocalized volume per unit area in *Wfs1* knockout mice (*Wfs1* knockout: 0.3921 ± 0.121 vs. WT: 1.000 ± 0.242; *p* = 0.0527; Figure 5D). Quantification of the percentage of PSD95 volume colocalized revealed an approximately 50% reduction in *Wfs1* knockout mice (*Wfs1* knockout: 23.80 ± 6.027 vs. WT: 44.60 ± 7.350; *p* = 0.0514; Figure 5E). The percentage of SYP volume colocalized showed no significant difference (*Wfs1* knockout: 16.55 ± 3.679 vs. WT: 19.16 ± 1.905; Figure 5F). Taken together, these results demonstrate that synaptic alterations are present at 4 months of age and progress to significant loss of synaptic connections by 7 months.

### 3.5 No sign of reactive gliosis in retina or optic nerve of Wfs1 knockout mice

**Figure 6:**
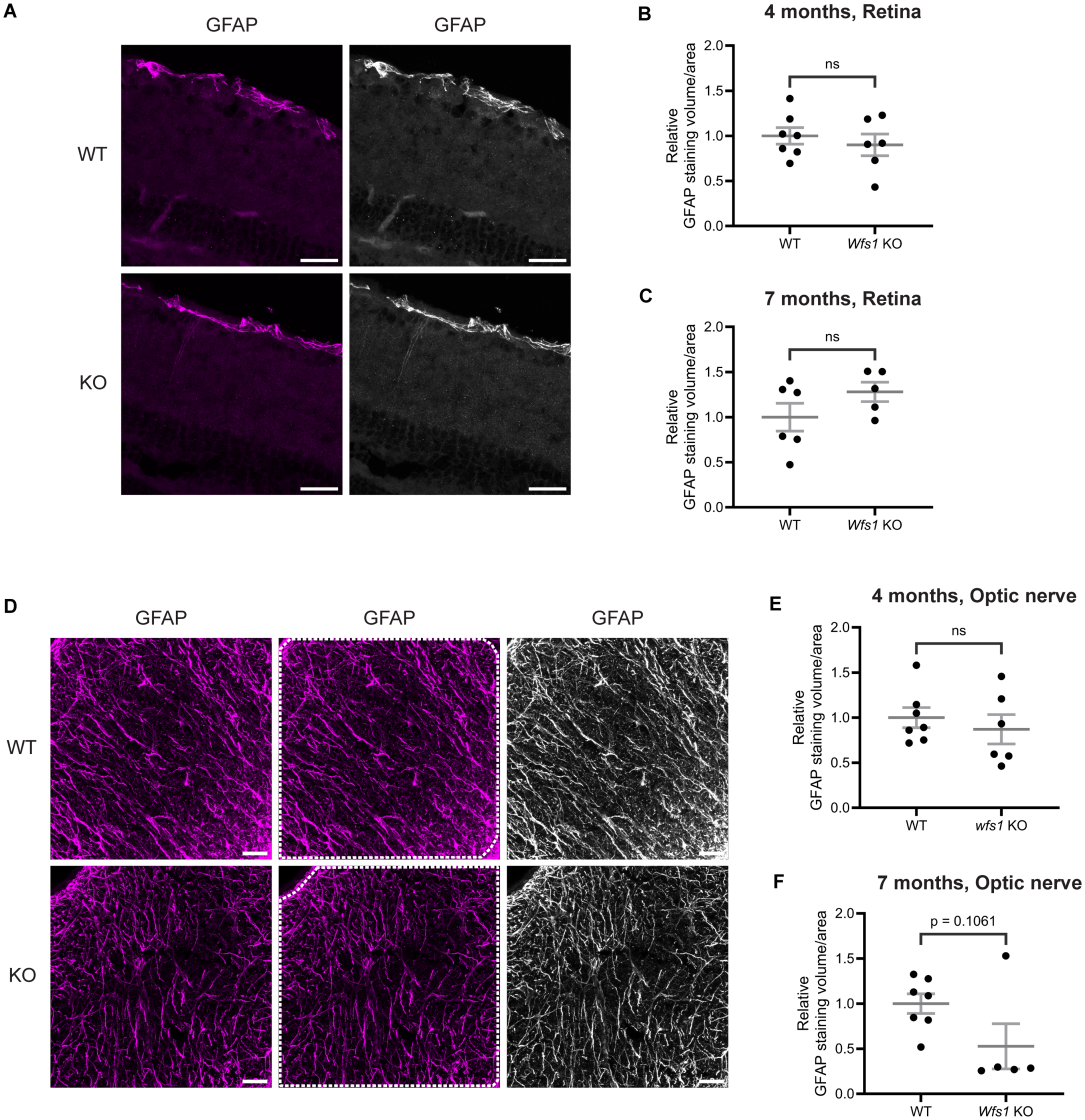
Elevated reactive gliosis was not evident in retina and optic nerve of Wfs1 KO mice. **(A)** Retinal sections were immunostained with an anti-GFAP antibody (magenta). Representative confocal images from 7-month-old WT (top) and Wfs1 KO (bottom) retinas are shown. Scale bar, 20 μm. Quantification of relative GFAP-positive staining volume in retinal sections from **(B)** 4-month **(C)** 7-month-old mice. Data points represent normalized values ± SEM relative to WT mean. Statistical analysis was performed using a two-tailed unpaired Student’s *t*-test with Welch’s correction. *n* ≥ 5. ns not significant. **(D)** Optic nerve sections were immunostained with an anti-GFAP antibody (magenta). Representative confocal images from 7-month-old WT (top) and Wfs1 KO (bottom) retinas are shown. Scale bar, 25 μm. Quantification of relative GFAP-positive staining volume in optic nerve sections from **(E)** 4-month **(F)** 7-month-old mice. Data points represent normalized values + SEM relative to WT mean. Statistical analysis was performed using a two-tailed unpaired Student’s *t*-test with Welch’s correction **(E)** and Mann–Whitney test **(F)**. *n* ≥ 5. ns - not significant.

To determine whether synaptic alterations were accompanied by glial activation, retinal sections were labeled with anti-GFAP. GFAP staining was largely restricted to the GCL and RNFL in both WT and *Wfs1* knockout mice at 7 months of age, indicating no extended glial processes (Figure 6A). Quantification of GFAP staining volume at 7 months showed a small, but non-significant, increase in *Wfs1* knockout mice (WT: 1.000 ± 0.154 vs. *Wfs1* knockout: 1.281 ± 0.107; Figure 6C); the 4-month group showed comparable values (WT: 1.000 ± 0.091 vs. *Wfs1* knockout: 0.9003 ± 0.120; Figure 6B). A similar pattern was observed for GFAP mean intensity in retinas at both 4 and 7 months of age (Supplementary Figures S2A–C).

Optic nerve sections from 4- and 7-month-old mice were also labeled with anti-GFAP. No sign of gliosis was observed in *Wfs1*-knokcout optic nerves at 7 months (Figure 6D). Strikingly, quantification of GFAP staining volume revealed an approximately 50% reduction in *Wfs1* knockout mice (WT: 1.000 ± 0.108 vs. Wfs1 knockout: 0.5276 ± 0.250; Figure 6F), while the 4-month group showed comparable values (WT: 1.000 ± 0.112 vs. *Wfs1* knockout: 0.8716 ± 0.162; Figure 6E). A similar pattern was observed for GFAP mean intensity in optic nerves at both ages (Supplementary Figures S2B–D). Collectively, these results indicate an absence of reactive gliosis in both retina and optic nerve of *Wfs1* knockout mice at 4 and 7 months of age.

### 3.6 Significant axonal loss in the optic nerve of Wfs1 knockout mice at 7 months

**Figure 7:**
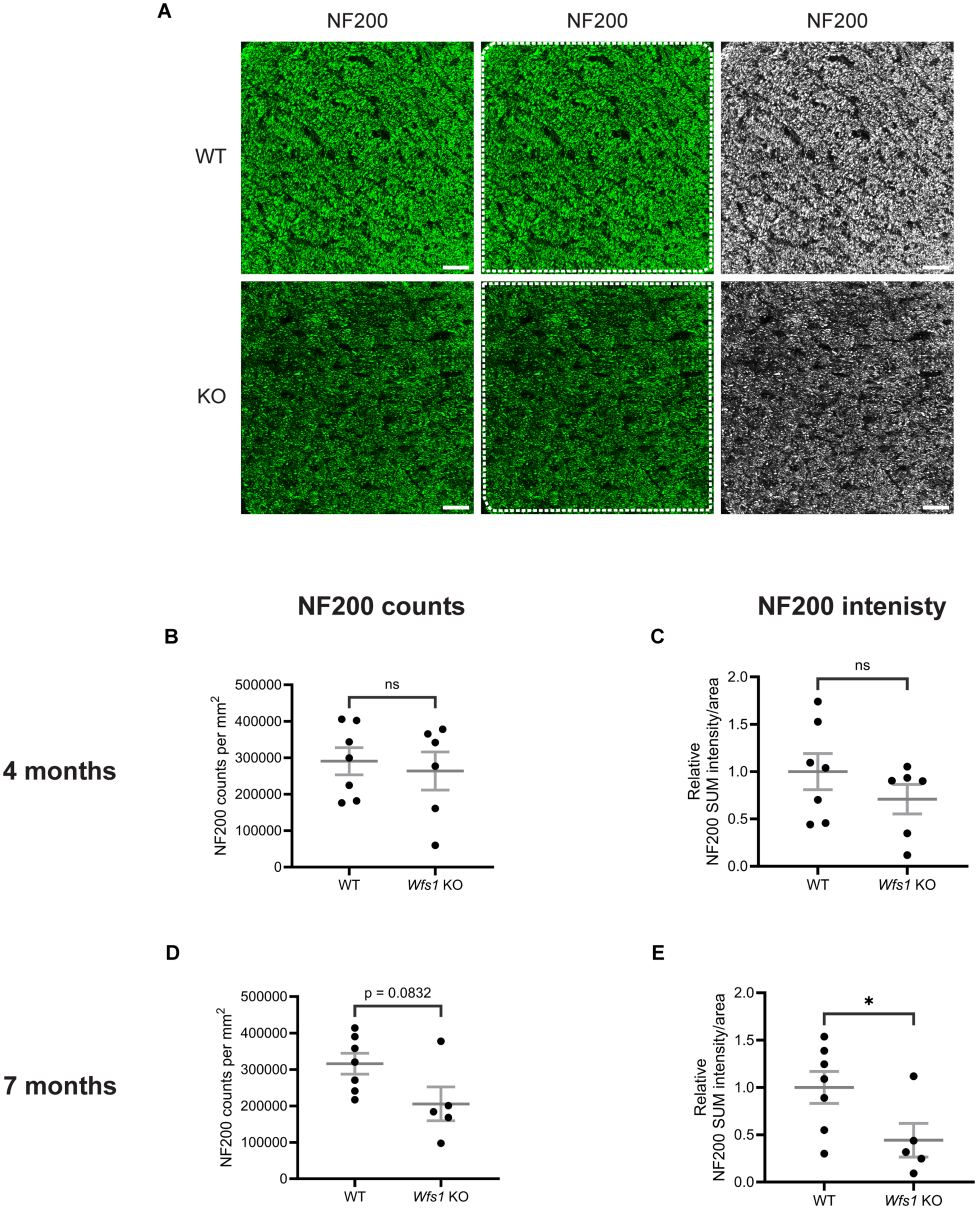
Axonal loss was observed in Wfs1 KO mouse model. **(A)** Mouse optic nerve sections were immunolabeled with an anti-NF200 antibody (green). Representative confocal images obtained for 7-month-old WT (top) and Wfs1 KO (bottom) mice are shown. Scale bar, 25 μm. Quantification of **(B)** axonal counts per mm^2^ and **(C)** NF200 staining volume per unit ROI area in the optic nerve sections of 4 month old mice. Quantification of **(D)** axonal counts per mm^2^ and **(E)** NF200 staining volume per unit ROI area in the optic nerve sections of 7 month old mice. Axonal counts were presented as mean ± SEM. NF200 SUM intensity data points were presented as normalized values ± SEM relative to the mean of WT group. Statistical analysis was performed by two-tailed, unpaired Student’s *t*-test with Welch’s correction. *n* ≥ 5. *p.

To evaluate axonal loss, a hallmark of optic atrophy, optic nerve transverse sections were immunolabeled with anti-neurofilament 200 (NF200) at 4 and 7 months of age. NF200 labeling was visibly reduced in *Wfs1* knockout animals at 7 months compared to WT controls (Figure 7A). Quantification of NF200 counts for the 7-month group yielded an average of 315,838 ± 28,571 per mm^2^ in WT compared to 205,618 ± 46,408 per mm^2^ in *Wfs1* knockout mice, a trend that approached significance (*p* = 0.0832; Figure 7D). Quantification of NF200 sum intensity per unit area showed a significant decrease in *Wfs1* knockout animals (WT: 1.000 ± 0.1698 vs. *Wfs1* knockout: 0.4428 ± 0.1778; *p* = 0.048; Figure 7E). In addition to this, quantification of the relative NF200-staining area fraction revealed a significant reduction, approximately 65%, in *Wfs1* knockout animals (WT: 1.00 ± 0.238 vs. *Wfs1* knockout: 0.35 ± 0.059; *p* = 0.033; Supplementary Figure S3). However, quantification of relative mean intensity showed a reduction in *Wfs1* KO mice compared to WT mice, but did not reach statistical significance (Data not shown).

In the 4-month group, NF200 counts were comparable (WT: 290,577 ± 37,146; *Wfs1* knockout: 263,909 ± 52,276; Figure 7B), and NF200 sum intensity showed only a modest, non-significant reduction (WT: 1.000 ± 0.1910; *Wfs1* knockout: 0.7090 ± 0.1553) (Figure 7C). These results demonstrate progressive, age-dependent axonal loss that is significant at 7 months in *Wfs1* knockout mice.

### 3.7 No sign of demyelination in Wfs1 knockout model

**Figure 8:**
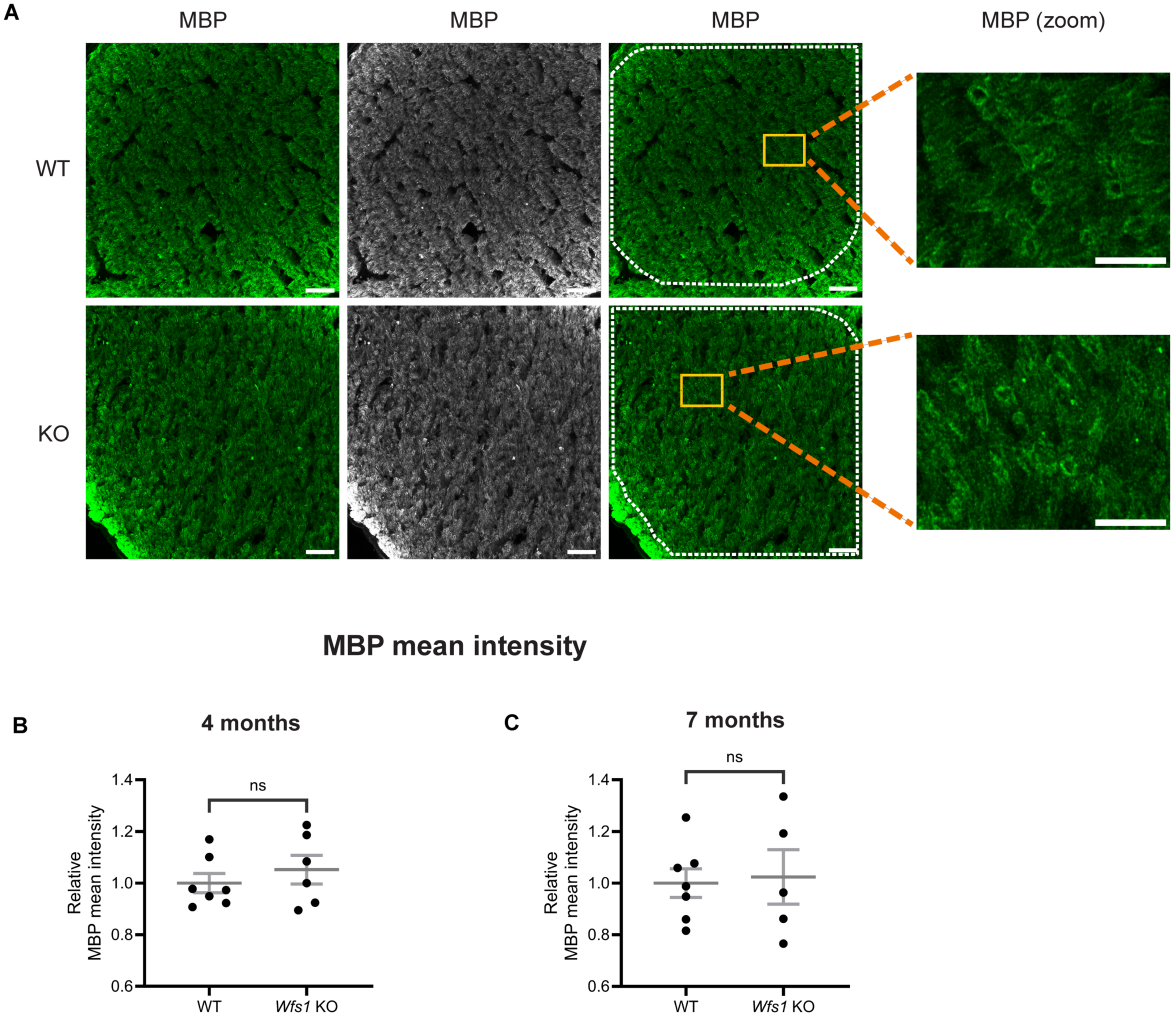
No evidence of demyelination was detected in optic nerves of Wfs1 KO mice at 4 and 7 months. **(A)** Optic nerve sections were immunostained with an anti-MBP antibody (green). Representative confocal images from 7-month-old WT (top) and Wfs1 KO (bottom) mice are shown along with the corresponding high-magnification zoom of the boxed area. Scale bar, 25 μm (inset scale bar, 10 μm). Quantification of MBP fluorescence mean intensity in optic nerve sections from **(B)** 4-month **(C)** 7-month old animals. Data are presented as normalized values ± SEM relative to the WT mean. Statistical analysis was performed using a two-tailed unpaired Student’s *t*-test with Welch’s correction. *n* ≥ 5. ns - not significant.

To determine whether axonal loss was accompanied by demyelination, optic nerve sections were immunostained with anti-myelin basic protein (MBP) at 4 and 7 months of age. MBP staining showed no difference in myelination between WT and *Wfs1* knockout animals at either age (Figure 8A). Quantification of MBP mean intensity confirmed no significant difference at 4 months (WT: 1.000 ± 0.037; *Wfs1* knockout: 1.052 ± 0.056) (Figure 8B) or 7 months (WT: 1.000 ± 0.056; *Wfs1* knockout: 1.024 ± 0.105) (Figure 8C). These results clearly indicate an absence of demyelination in the *Wfs1* knockout mouse model, even with age progression.

## 4 Discussion

**Figure 9:**
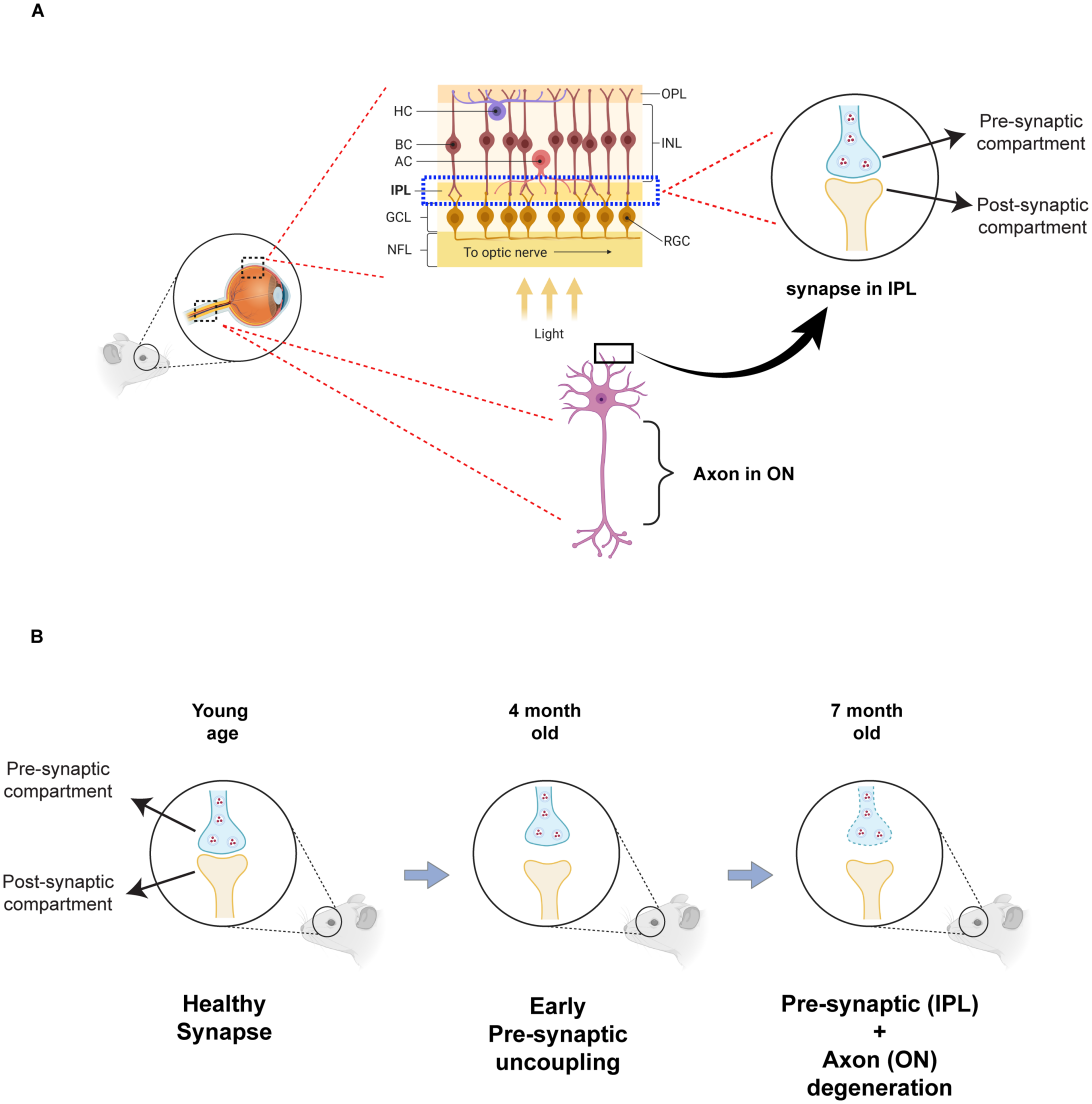
Schematic model of progressive synaptic loss in retina of Wfs1 KO mouse model. **(A)** Schematic representation of retinal layers illustrating the location of synapses within the inner plexiform layer (IPL) and the trajectory of retinal ganglion cell (RGC) axons projecting through the optic nerve. Pre- and post-synaptic compartments are shown in apposition under healthy conditions. **(B)** Model summarizing age-dependent synaptic alterations in Wfs1-knockout mice. At young ages, synapses appear intact with closely aligned pre- and post-synaptic elements. By 4 months, early pre-synaptic uncoupling or spatial de-alignment is evident, reflected by reduced synaptophysin colocalization with PSD95. By 7 months, pronounced pre-synaptic degeneration occurs, accompanied by decreased synaptophysin levels, reduced synaptic colocalization, and concurrent axonal loss in the optic nerve. Created with BioRender.com. OPL, outer plexiform layer; INL, inner nuclear layer; HC, horizontal cell; BC, bipolar cell; AC, amacrine cell; IPL, inner plexiform layer; GCL, ganglion cell layer; RGC, retinal ganglion cell; NFL, nerve fiber layer.

Wolfram syndrome is a rare genetic disease characterized by early-onset antibody-negative diabetes mellitus, optic atrophy, and neurodegeneration. Preclinical models in mice, rats, and zebrafish have been developed to model Wolfram syndrome and have been shown to exhibit visual defects, including RGC loss, axonal loss, myelin degeneration, and reactive gliosis (Bonnet Wersinger et al., 2014; Waszczykowska et al., 2020; Rossi et al., 2023; Ahuja et al., 2024; Seppa et al., 2019; Plaas et al., 2017; Jagomae et al., 2021; Cairns et al., 2021). However, no previous studies in these models investigated synaptic and dendritic connections. *In vitro* evidence from cerebral organoids and iPSC-derived neurons indicates that *WFS1*-deficient neurons exhibit disrupted synapses and altered neurites (Yuan et al., 2023; Pourtoy-Brasselet et al., 2021), underscoring the need for *in vivo* investigation. The present study, for the first time, reveals that synaptic alterations precede axonal loss and represent the earliest phenotype in optic atrophy of the *Wfs1* knockout mouse model (Figure 9).

The IPL of the mammalian retina harbors the synapses and dendrites of RGCs, which form connections with bipolar and amacrine cells from the INL. Our study reveals that the percentage of SYP colocalized with PSD95 is significantly decreased in *Wfs1* knockout mice compared to WT at 4 months of age, despite no significant changes in the levels of pre- or post-synaptic compartments at that age, likely reflecting uncoupling of presynaptic compartments. At 7 months, mutant mice exhibit loss of presynaptic compartments (decreased SYP mean intensity and volume), reduced overall colocalized volume, and a decreased percentage of PSD95 colocalized with SYP, indicating progressive loss of functional synapses. This pattern of early presynaptic uncoupling followed by eventual compartment loss at advanced disease stages indicates a key role for presynaptic failure in the mechanism of Wolfram syndrome. Notably, similar results were reported in cerebral organoids, where *WFS1*-deficient neurons showed decreased colocalization of Synapsin 1 with PSD95 and reduced synapse density (Yuan et al., 2023). Likewise, WFS1 suppression in primary cortical neurons reduced synaptic density at DIV19 but not at earlier developmental stages (Cagalinec et al., 2016). This early synaptic dysfunction, established in this study, may explain the visual defects observed well before axonal loss in both mouse and rat models (Ahuja et al., 2024; Jagomae et al., 2021). Furthermore, presynaptic failure is well documented as an early event in Alzheimer’s disease and dominant optic atrophy (Anschuetz et al., 2024; Atamena et al., 2023; Bell and Claudio Cuello, 2006; Masliah et al., 2001; Sze et al., 1997), while reduction of PSD95 has been shown to occur at a later stage in Alzheimer’s disease (Shao et al., 2011), consistent with the relatively stable postsynaptic compartment observed here in early-stage disease. A limitation of this strategy, employed in this study, is the difficulty in achieving a higher signal-to-noise ratio, which could be overcome by employing super-resolution microscopy, such as STED, in future studies to investigate sub lamina, ON- and OFF- specific changes in IPL. Importantly, our findings here are based on structural and molecular readouts of pre- and post-synaptic compartments; future studies incorporating electrophysiology, calcium-imaging approaches, and sublamina-specific analyses will be essential to determine how these structural changes translate into functional deficits in *Wfs*1-deficient retinas.

Further retinal investigation confirms no RGC loss and no dendritic loss in *Wfs1* knockout mice in this study. Using both Brn3a (which labels approximately 85% of murine RGCs) (Galindo-Romero et al., 2011) and RBPMS (which labels nearly all RGCs) (Rodriguez et al., 2014), we confirm no RGC loss at 7 months of age. We show no gross dendritic loss in the IPL at either 4 or 7 months, which contrasts with findings from human iPSC-derived neurons, where neurite outgrowth was impaired (Pourtoy-Brasselet et al., 2021). However, a limitation of this study is that confocal imaging captures gross morphological changes but lacks the resolution to detect subtle alterations in dendritic arborization. To evaluate dendritic alterations more accurately, genetic neuronal labeling or DiOlistic labeling, combined with Sholl analysis, dendritic length, and branching, would reveal if there is any change. Also, sublamina-specific quantification would provide a more precise assessment of localized structural changes within the IPL, as has been described in dominant optic atrophy (Williams et al., 2010) and glaucoma (El-Danaf and Huberman, 2015). Another limitation concerns the analysis time window, as some *Wfs1*-knockout mice develop spontaneous eye rupture after 7 months of age, precluding analysis of older animals for ethical reasons. The underlying mechanism of this phenotype remains unclear.

In this study, we have not shown evidence of statistically significant reactive gliosis in either the retina or the optic nerve. However, we observe almost 50% reduction of GFAP staining volume in the optic nerve of *Wfs1* knockout mice compared to the WT mice at 7 months of age whereas the retina shows a mild, non-significant increase in GFAP staining volume in the knockout animals with no clear differences in the GFAP staining at 4 months. This anatomical discrepancy can be attributed to the heterogeneous distribution of macroglia; while the retina (gray matter) housing both Muller cells and astrocytes, the optic nerve (white matter) is completely devoid of Muller cells but mainly fibrous astrocytes, which are different due to their structural and functional properties (Cullen and Sun, 2023). While retinal GFAP levels showed a nonsignificant upward trend, the unexpected reduction of GFAP in the optic nerve points towards the role of fibrous astrocytes in white matter in this knockout model. A recent study reported increased GFAP staining in the trigeminal sensory nucleus (TSN) of *Wfs1*-deficient mice at 8 months of age (Tulva et al., 2025). However, although the authors did not discuss it, the published image reveals a clear reduction in GFAP staining in the spinal tract of the trigeminal nerve (white matter) surrounding the TSN (gray matter). This raises concerns about the role of astrocytes in white matter. While no previous studies investigated the reactive gliosis state in the optic nerve, a few studies revealed the gliosis state in the retina of the *Wfs1* knockout mouse model. Increased gliosis in the retina in *Wfs1* knockout mice was shown before (Rossi et al., 2023). This contradictory result, not having a significant gliosis state in the retina, can be attributed to the genetic background of the mice, which was shown to impact the severity of the phenotype (Atamena et al., 2023). However, our findings are consistent with another study that showed reduced GFAP intensity in the retina of *Wfs1* knockout mice (Waszczykowska et al., 2020). Furthermore, it was demonstrated that *WFS1*-deficient cerebral organoids had reduced number of astrocytes (Yuan et al., 2023). It is also noteworthy that two recent studies identified a reduced number of oligodendrocyte precursor cells (Ahuja et al., 2024) and functional defects in MBP-positive oligodendrocytes (Rossi et al., 2023) in the optic nerve of the *Wfs1* knockout mice. Altogether, our findings of reduced gliosis in the optic nerve indicate impaired astrocytic activity in white matter in *Wfs1* knockout mice and warrant further investigation to determine whether this change is a cause or a consequence of disease progression.

Regarding axonal integrity, we observe an approximately 40% reduction in NF200 intensity in *Wfs1* knockout mice compared to WT at 7 months, with no significant loss at 4 months. This result is consistent with previous reports of axonal loss without concurrent RGC degeneration (Rossi et al., 2023), while the later onset relative to that study may be attributed to differences in genetic background (Atamena et al., 2023). No demyelination is detected at either age, though we cannot exclude subtle abnormalities that would require transmission electron microscopy (TEM) analysis to detect.

This study, for the first time, identifies synaptic alterations as the earliest disease phenotype in the optic atrophy of Wolfram syndrome, preceding axonal loss in *Wfs1* knockout mice. As the disease progresses, presynaptic failure drives the overall loss of functional synapses, mirroring the early presynaptic pathology described in Alzheimer’s disease and dominant optic atrophy and underscoring the presynaptic machinery as a therapeutically tractable target in Wolfram syndrome. We further identify reduced astrocytic reactivity in the optic nerve as a novel disease feature, pointing to white-matter astrocyte failure as an unexplored axis of disease progression. Together, these findings redefine the therapeutic window for vision loss in Wolfram syndrome: rather than acting after axonal degeneration or ganglion cell death is established, interventions designed to preserve synaptic integrity and support white-matter astrocyte function at the pre-degenerative stage offer a tractable strategy to delay or prevent irreversible vision loss, with broader implications for neurodegenerative disorders that share an early presynaptic pathology.

## Data Availability

The datasets presented in this article will be made available upon reasonable request. Requests to access the datasets should be directed to urano@wustl.edu.
