# Supplementary material for: Synaptic alterations are preceding the axonal loss in optic atrophy of Wolfram syndrome mouse model
Source: Front Neurosci. 2026 May 22;20:1838257. doi: 10.3389/fnins.2026.1838257 (PMC13236678; doi:10.3389/fnins.2026.1838257)
Supplement: Supplementary file 1 [file Data_Sheet_1.PDF]

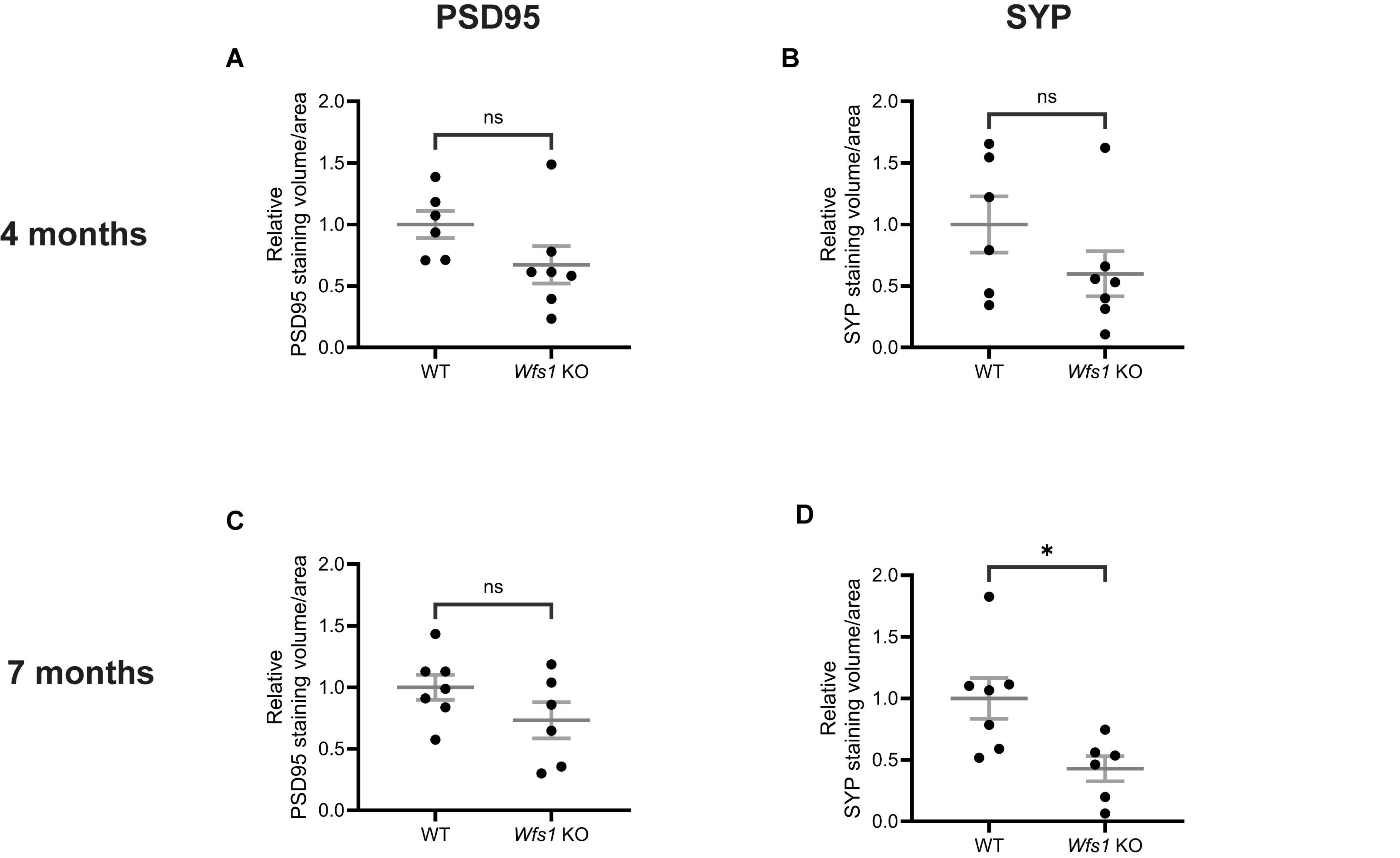

Supplementary Figure 1. Quantification of synaptic proteins in *Wfs1* KO mice.
Relative quantification of (A,C) PSD95 and (B,D) synaptophysin staining volume in 4 (A and B) and 7 (C and D) month old *Wfs1* KO mice compared to WT mice. Data are presented as normalized values ± SEM relative to WT mean. Statistical comparisons were performed using a two-tailed unpaired Student's t-test with Welch's correction (A, C, D) and Mann-Whitney test (B). n ≥ 6. $^{*}p < 0.05$. ns - not significant.

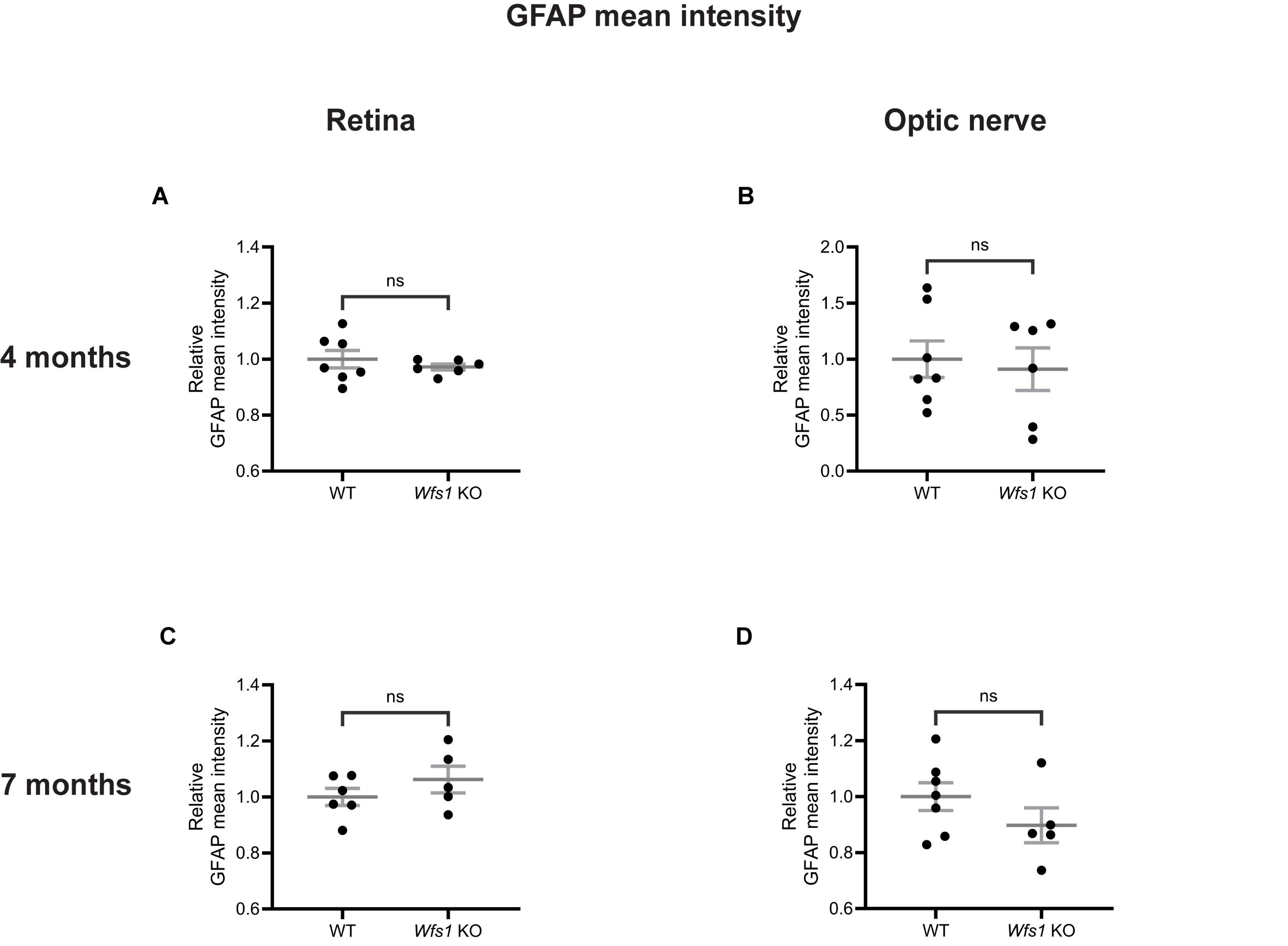

Supplementary Figure 2. Quantification gliosis state in retina and optic nerve of *Wfs1*-KO mouse model.
Relative quantification of GFAP staining mean intensity in retina (A and C) and optic nerve (B and D) of 4 (A and B) and 7 (C and D) month old *Wfs1* KO mice compared to WT mice. Data represent normalized values ± SEM relative to WT mean. Statistical comparisons were performed using a two-tailed unpaired Student's t-test with Welch's correction. n ≥ 5. ns - not significant.

7 month, NF200 staining area

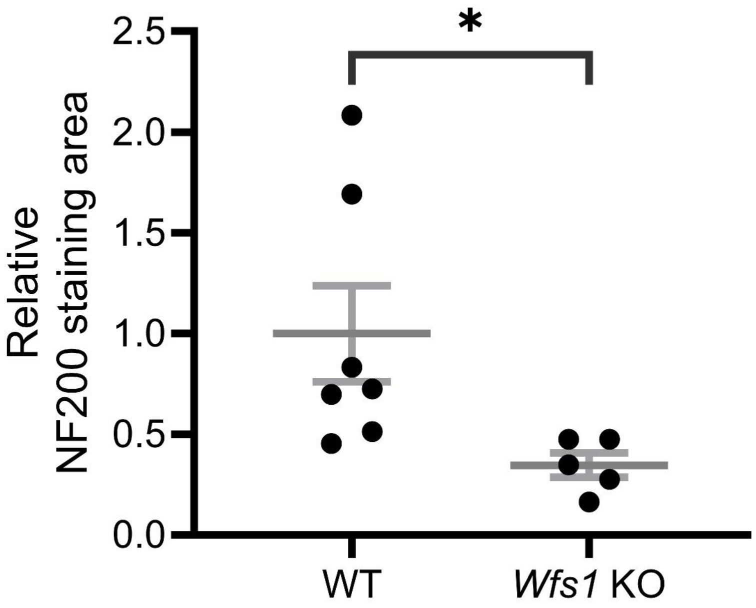

Supplementary Figure 3. Quantification of NF200 staining area in optic nerve section of *Wfs1* KO model. Relative quantification of NF200 staining area in optic nerve sections of 7 month old *Wfs1* KO mice compared to WT mice. Data points represent normalized values ± SEM relative to WT mean. Statistical comparison was performed using a two-tailed unpaired Student's t-test with Welch's correction. n ≥ 5. *p<0.05.
